# User Interaction in Semi-Automatic Segmentation of Organs at Risk: a Case Study in Radiotherapy

**DOI:** 10.1007/s10278-015-9839-8

**Published:** 2015-11-09

**Authors:** Anjana Ramkumar, Jose Dolz, Hortense A. Kirisli, Sonja Adebahr, Tanja Schimek-Jasch, Ursula Nestle, Laurent Massoptier, Edit Varga, Pieter Jan Stappers, Wiro J. Niessen, Yu Song

**Affiliations:** Faculty of Industrial Design Engineering, Delft University of Technology, Landbergstraat 15, 2628CE Delft, The Netherlands; Aquilab, Loos-les-Lille, Lille, France; Department of Radiation Oncology, University Medical Center Freiburg, Freiburg, Germany; Department of Medical Informatics, Erasmus MC, Rotterdam, The Netherlands; Faculty of Applied Science, Delft University of Technology, Delft, The Netherlands

**Keywords:** Radiotherapy, Organs at risk, Semi-automatic segmentation, Human-computer interaction, Evaluation, Correlations

## Abstract

Accurate segmentation of organs at risk is an important step in radiotherapy planning. Manual segmentation being a tedious procedure and prone to inter- and intra-observer variability, there is a growing interest in automated segmentation methods. However, automatic methods frequently fail to provide satisfactory result, and post-processing corrections are often needed. Semi-automatic segmentation methods are designed to overcome these problems by combining physicians’ expertise and computers’ potential. This study evaluates two semi-automatic segmentation methods with different types of user interactions, named the “strokes” and the “contour”, to provide insights into the role and impact of human-computer interaction. Two physicians participated in the experiment. In total, 42 case studies were carried out on five different types of organs at risk. For each case study, both the human-computer interaction process and quality of the segmentation results were measured subjectively and objectively. Furthermore, different measures of the process and the results were correlated. A total of 36 quantifiable and ten non-quantifiable correlations were identified for each type of interaction. Among those pairs of measures, 20 of the contour method and 22 of the strokes method were strongly or moderately correlated, either directly or inversely. Based on those correlated measures, it is concluded that: (1) in the design of semi-automatic segmentation methods, user interactions need to be less cognitively challenging; (2) based on the observed workflows and preferences of physicians, there is a need for flexibility in the interface design; (3) the correlated measures provide insights that can be used in improving user interaction design.

## Introduction

In radiotherapy planning, three fundamental axioms are often applied [1]: (1) an increased dose to the tumor normally improves the local control; (2) improving local control of tumors improves overall cure rate, as it prevents metastatic spread from local recurrence; and (3) sparing normal tissues decreases the side effects of radiotherapy. Thus, to maximize the delivery of radiation dose to the tumor while sparing the normal tissues, accurate segmentation of tumor and organs at risk on medical images is a prerequisite.

Manual segmentation performed by experts is often used as the reference standard in radiotherapy planning [2]. Using manual segmentation methods, physicians segment the organs by drawing contours on medical images slice by slice based on their clinical knowledge. The process is generally time consuming, demands high workload due to intensive human-computer interactions (HCI) and lacks reproducibility [3, 4].

To overcome the limitations of manual segmentation, automated segmentation methods have been introduced. These methods have shown to be an effective solution for various applications [5, 6] as they are usually faster than manual segmentation methods, and require no or few user interactions during the segmentation process [7–9]. However, the outcomes are sensitive to image quality, which highly depends on the acquisition protocols [10]. In many cases, automatic segmentation methods can only be applied successfully within pre-defined conditions and extensive post-processing is often needed. For instance, Wu et al. [6] identified that their automatic segmentation method performed well for large organs, while manual corrections were often required for smaller organs. Sims et al. [11] also concluded that careful review and manual editing were required for most segmentation results obtained by automatic methods.

**Fig. 1:**
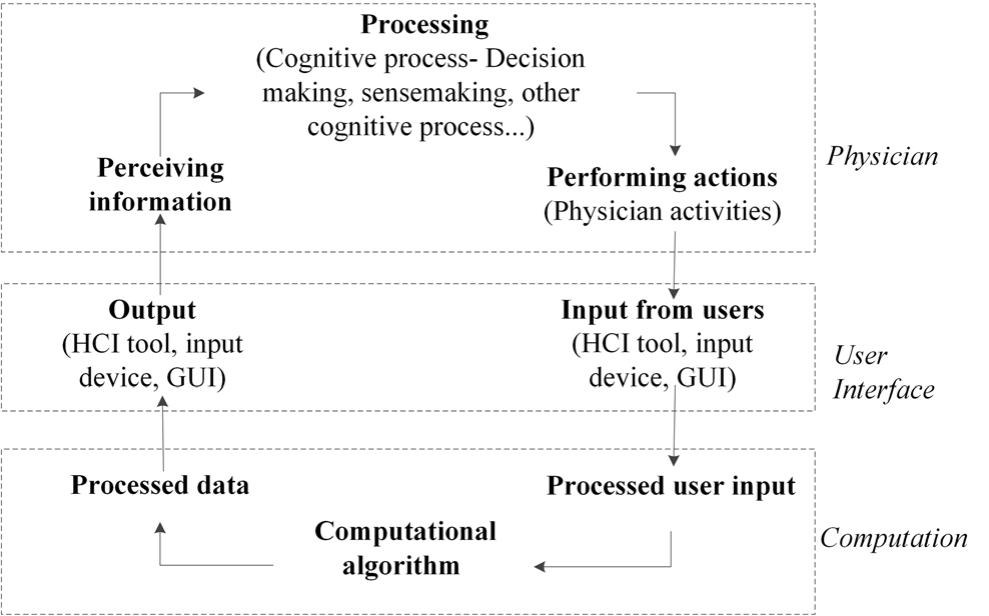
Information flow of human-computer interaction in a SAS method

By engaging physicians in between the computational algorithm, semi-automatic segmentation (SAS) methods were developed. SAS are partially supervised automatic methods and they provide solutions by combining physicians’ expertise and the computing power of the computer [12–14]. Figure 1 presents a typical information flow of the SAS method [15, 16]. The flow starts from a physician, who first perceives the information on the dataset to get familiarized with the case. After acquiring the information from the dataset, the physician decides on the next step in the segmentation process and performs the required *action*. Here the term *action* refers to the physical activities performed by the physician such as moving their hand to choose the input device, scrolling the mouse button to select the desired plane/tool, pressing the zoom-in/out button, initializing the segmentation by drawing contours, and positioning their hand in case of gesture interaction. *Actions* performed by the physician are interpreted by software via the graphical user interface. Once confirmed, the medical images are processed by a computational algorithm(s) utilizing the input(s), and the output data is displayed on the user interface. This process iterates until a satisfied result is achieved.

Effectiveness and efficiency of a SAS method depend on the proper combination of physicians’ expertise and the capability of the computational method [17]. Though physicians play a crucial role in the segmentation process, research on the development of SAS methods has mainly focused on the computational part [18]. The cognitive aspects of physicians and designed human-computer interaction in the segmentation process have only been addressed in few works [16, 19, 20].

In this paper, we investigate the effects of user interaction in SAS methods regarding the segmentation of organs at risk for radiotherapy planning in order to propose suggestions for further improvements. To achieve this, two SAS methods with the same workflow but two different types of user interaction were developed. A case study was conducted where physicians were asked to segment five organs using the two SAS methods. In the investigation, both human-computer interaction process and the quality of the segmentation results were measured subjectively and objectively. To aid in the interpretation of the results, we identified correlations between the measurements obtained. In this way, we were able to distinguish the effectiveness and efficiency of user interactions in various steps of the SAS methods. Finally, suggestions regarding the design of user interactions in SAS methods are proposed based on these findings.

The remainder of this paper is organized as follows: In section 2, research regarding the workflow of SAS methods, HCI in SAS methods and evaluation methods are reviewed. The two SAS methods used in this research are introduced in section 3 with the focus on the workflow and the design of HCIs. Section 4 lists the setup and protocol of the experiment. Experimental results are analyzed and presented in section 5. The findings in those results are discussed in section 6 where suggestions for the design of user interactions are presented as well. Finally, conclusions are drawn in section 7.

## Literature Review

In a SAS method, the workflow is often designed based on the characteristic of computational algorithms and available HCI devices. A review of the literature indicates that three different types of workflow are often implemented [16]. In the first type, a physician initializes the segmentation algorithm and depending on the outcome, manual editing may be performed until a satisfactory result is achieved [21]. The second one is similar to the first: a physician first initializes the segmentation algorithm and if the result is not satisfactory, instead of editing the result manually, he/she may re-initialize the segmentation algorithm [19]. In the third type, the physician modifies the obtained result in a local region such that only the area where the segmentation is not satisfactory is indicated by the physician and is corrected automatically using various algorithm [22]. In the workflow of a SAS method, mouse, keyboard, and screen are the most often used human-computer interaction devices. However, there are many other devices which may facilitate this process. For instance, Harders et al. [20] evaluated the value of haptic feedback in a multimodal setting and found that the used approach is only applicable to linear structures. Sherbondy et al. [23] evaluated user input devices such as trackball, pen-tablet, jog-shuttle wheel, and mouse. They found that the pen-tablet in two distinct configurations performed faster than the mouse and trackball in a simulated angiography localization task. Besides those devices, a different approach to interactive segmentation was introduced by Sadeghi et al. [24], who used eye gaze to guide the segmentation. However, accurate placement of strokes might be strenuous on the eyes for complicated medical images.

Using HCI devices, physicians may select different HCI tools to perform interaction. Olabarriaga et al. [16] investigated HCI issues in 2D segmentation and one main focus was on the segmentation tools used, such as deform, edit boundary, and rectangle. Aselmaa et al. [25] concluded that in manual segmentation tasks, brush tool, 3D pencil, smart brush, and nudging were often used. Using these tools, physicians may perform different types of interactions such as fine tuning parameters, drawing lines, marking points, and drawing bounding boxes [26], to provide inputs to computational algorithms. Using HCI tools, various types of user input can be designed. Yang et al. [27] concluded from their study that the type of user input is an important factor that has to be taken into account as it also affects the outcome of the segmentation result. Hebbalaguppe et al. [28] compared three different types of user input for semi-automatic segmentation and identified the relations between them and the segmentation result. The Lazy Snapping work [26] integrated intuitive user interfaces, such as foreground/background strokes and boundary polygon editing, to emphasize the desirability of a limited amount of user inputs.

Another relevant aspect is the HCI patterns observed during the process of SAS methods. HCI patterns are a series of repetitive physical actions that are executed during segmentation, for instance, continuous zooming in and out, constant scrolling through a set of images, and constant alteration of window levels. These patterns are developed based on physicians’ clinical knowledge and personal preferences, and the outcome of segmentation is influenced by these patterns. A study conducted by Dalah et al. [29] proved that changes in window level settings during segmentation produced about 2 mm discrepancies in the outcomes. Other studies [30, 31] on HCI patterns also revealed the influence while performing certain tasks. Ju and Leifer [32] discussed that identifying the HCI patterns can be useful for designers to overcome the interaction design problems and help them leverage existing linguistic, sociological, or ethnographic techniques for designing better human-computer interaction.

In order to improve the usability of the input devices, tools, and types of user input, a proper usability evaluation of current designs is required. ISO 9241 part 11 [33] defines usability as “*the extent to which a product can be used by specified users to achieve specified goals with effectiveness, efficiency and satisfaction in a specified context of use*”. Here effectiveness refers to the degree of completeness and accuracy with which the work/goal is achieved. Efficiency refers to how much effort and how much time physicians spent to finish a task. Satisfaction denotes to what extent physicians are satisfied with efficiency and effectiveness of the task. Thus, in the usability evaluation of a SAS method, both the result and the process should be assessed to measure effectiveness, efficiency, and satisfaction.

A variety of usability evaluation methods have been used to detect the usability problems related to technology. They are the following: heuristic evaluation [34], cognitive walkthrough [35], cognitive task analysis [36, 37], think-aloud protocol [38], usability surveys [39], etc. Most of the usability studies include subjective and objective measures, some quantifying the HCI process, others quantifying the result. Among those studies, objective measures of the HCI process have gradually gained attention. For instance, Coen [40] evaluated the HCI input devices by measuring the number of mouse clicks, mouse movement, zooming, panning, scrolling, corrections, and related the interaction patterns regarding the segmentation result. A similar study in radiotherapy conducted by Kotani and Horii [41] compared interactions between the pen-tablet and the mouse. In their study, the error rate was a measure of the result and electromyography was a measure of the process. Hebbalaguppe et al. [29] assessed the cognitive workload by means of electroencephalogram signals. In their study, electroencephalogram signals were found to be correlated to attention, emotion, and decision-making of the users. Olabarriaga and Smeulders [16] evaluated the effectiveness of HCI by measuring the accuracy and reproducibility of the system. McGuinness and O’Connor [42] compared four interactive segmentation techniques by comparing users’ perception and the measurement result. Though considerable effort has been devoted to this area, the use of the subjective or/and objective measures in usability evaluation is still a challenging question [43, 44]. For instance, Hornbæk [45] concluded that identifying relations between the HCI process and the outcomes from the measurement are relevant direction for future research.

## Two Types of User Interaction in the Proposed SAS Methods

**Fig. 2:**
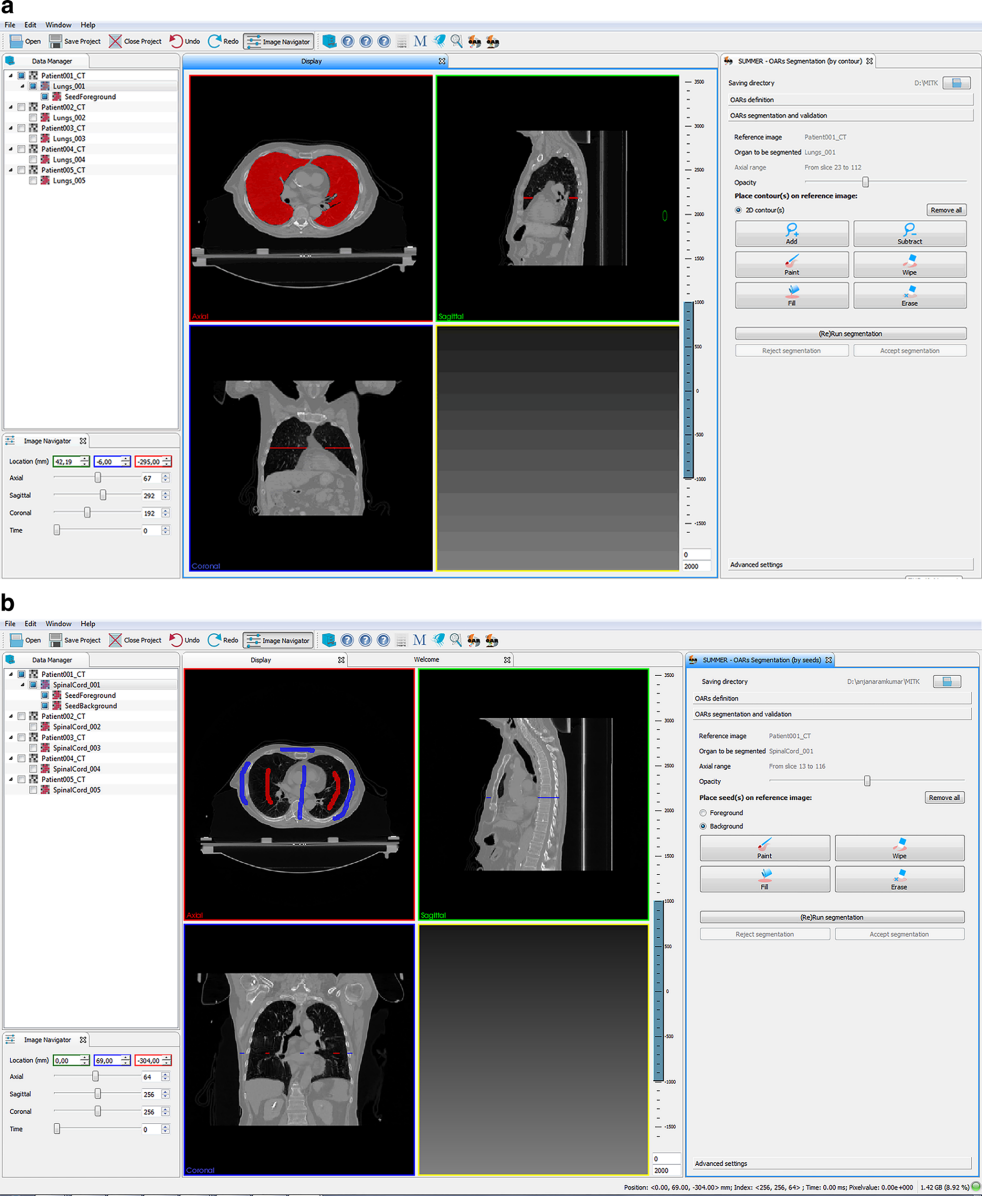
User interfaces of the proposed two SAS methods. **a** User Interface of the contour method **b** User Interface of the strokes method

In the proposed research, two SAS methods with the same workflow but different interactions were developed. The first SAS method, which is referred as the “contour” method, requires the physician to draw contours in a limited number of slices as shown in Fig. 2a and the algorithm then computes the segmented volume in 3D. Physicians are often familiar with the contour method, as it is used for segmentation in their clinical routine. Using this method, physicians were instructed to trace the boundary of the organ accurately on the slice they select. It is assumed that the interaction can be physically and mentally demanding for the physician. In this context physical demand refers to the laborious and time-consuming contouring. Mental demand refers to the task which involves considerable thinking and scrolling, in which the physician needs to be more focused.

The second SAS method is the “strokes” method which is designed to reduce the physical and mental demands of physicians. The physician draws strokes to indicate the foreground (as the two red strokes in Fig. 2b) that represents the region the physician wants to include as an organ and the background strokes (as the four blue strokes in Fig. 2b) that distinguishes the areas which should not be included in the organ contour. The algorithm then computes the segmentation volume. With strokes interaction, physicians may indicate the region of interest by drawing a line or placing some dots, and it is expected that the physical and mental demands are lower than using the contour method. However, compared to contour method, strokes method is not widely used in radiotherapy.

**Fig. 3:**
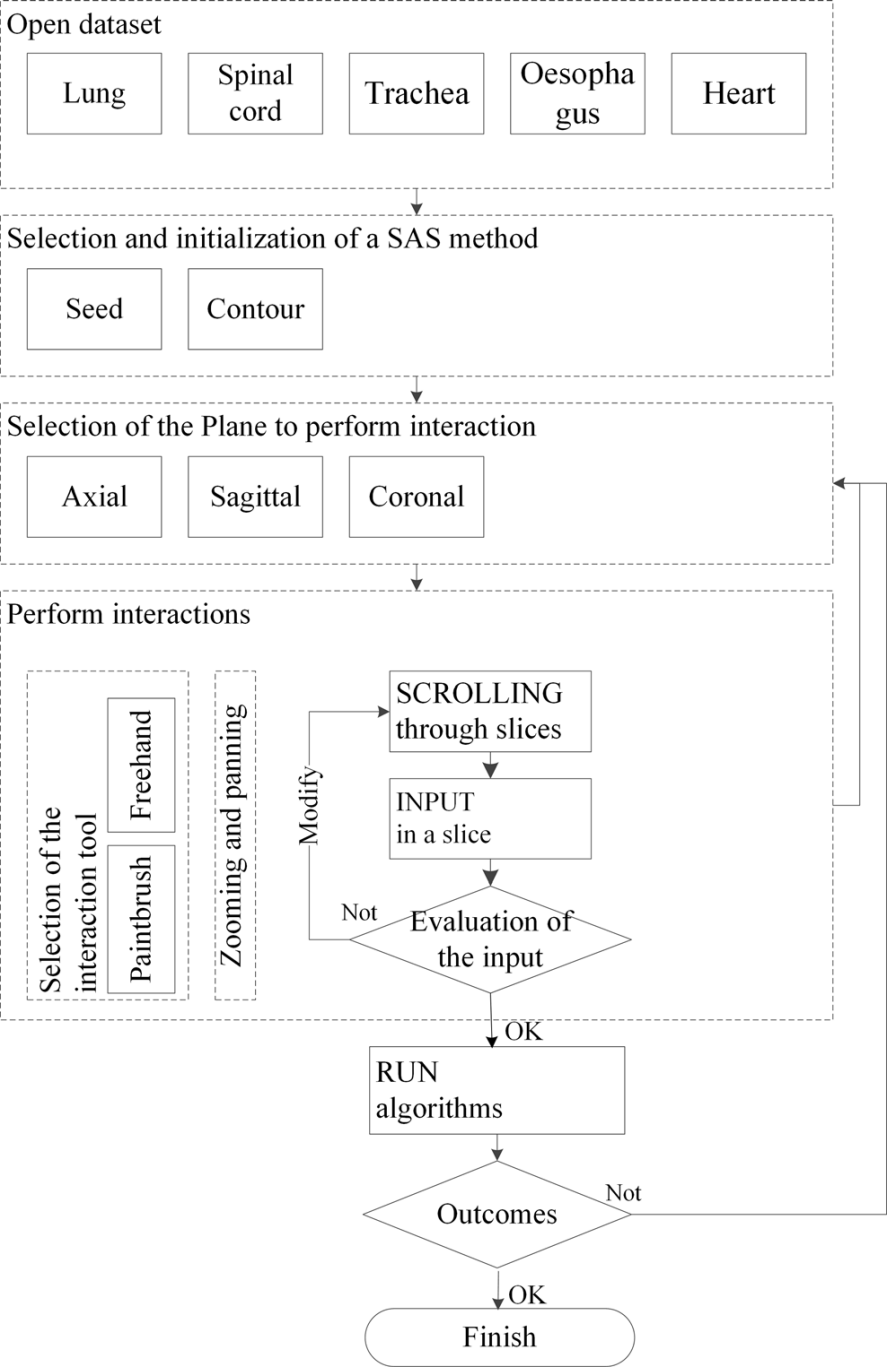
Workflow of the proposed SAS methods

In order to make a valid comparison of the effects of user interactions in using SAS methods, the second type of workflow presented in section 2 was adopted in both methods as Fig. 3. The reason for using this workflow is to maximally preserve the combined effects of HCI and the algorithm. If manual modifications were allowed, then the quality of the outcome would be hard to judge, as it would be unclear whether it was produced by the SAS method or manual modifications. In the workflow, after the physician loads a new dataset, he/she can choose either the contour or the strokes method to segment the organ. Physicians can perform actions on axial, sagittal, or coronal planes with the help of HCI tools. The physician may scroll through all the slices, provide certain input on the desired slices and modify until a satisfied input for the algorithm is achieved. Then the physician runs the algorithm with the provided input and evaluates the outcome. If the outcome is not satisfactory, the physician may re-define the inputs of the algorithm and re-run the segmentation process. Maximally five iterations for each organ were given to the physician and if the result is not satisfactory after the fifth iteration, the segmentation is considered to be unsuccessful.

A prototype of both SAS methods was developed as a plug-in on the medical imaging and interaction toolkit (MITK) platform, version 2013.09.0 [45]. For both SAS methods, a combination of graph-cut and watershed-based algorithms was developed by Dolz et al. [46, 47], and was implemented as the computational part in the prototype. Figure 2 shows screenshots of two methods in the prototype. The left window of the display contains the data manager, which allows the physician to select and view the dataset. The main rendering window is presented at the center with four quadrants, three of them displaying different orthogonal views. The bottom right quadrant shows the segmentation result as a 3D rendering. 2D HCI inputs can be performed in the axial, the coronal, and the sagittal view with a mouse. *Tools* which can be used for drawing and modifications are on the right side of the interface. In the contour method, a “free hand” tool can be selected by clicking the “add” button on the interface. Besides, physicians can also use a “paint” (paintbrush) tool, with adjustable brush size. In the strokes method, the accuracy requirement of the interaction is not high, thus the “paint” was the only tool that was provided.

Similar to the prototype developed by Heckel et al. [21], the prototype used in this research is designed in such a way that physicians can give their inputs in any orthogonal planes. Currently in clinical practice, physicians often use only axial view to give their inputs and the other views are often used to check if the segmentation result is satisfactory. By giving the freedom to draw in any orthogonal planes, physicians may choose the plane which requires few HCI. For instance, when segmenting the spinal cord, physicians can segment in the sagittal or coronal planes. It is expected that this design may reduce the number of user inputs, as well as the time taken for drawing the contours/strokes due to fewer slices.

## User Testing Setup and Protocol

**Fig. 4:**
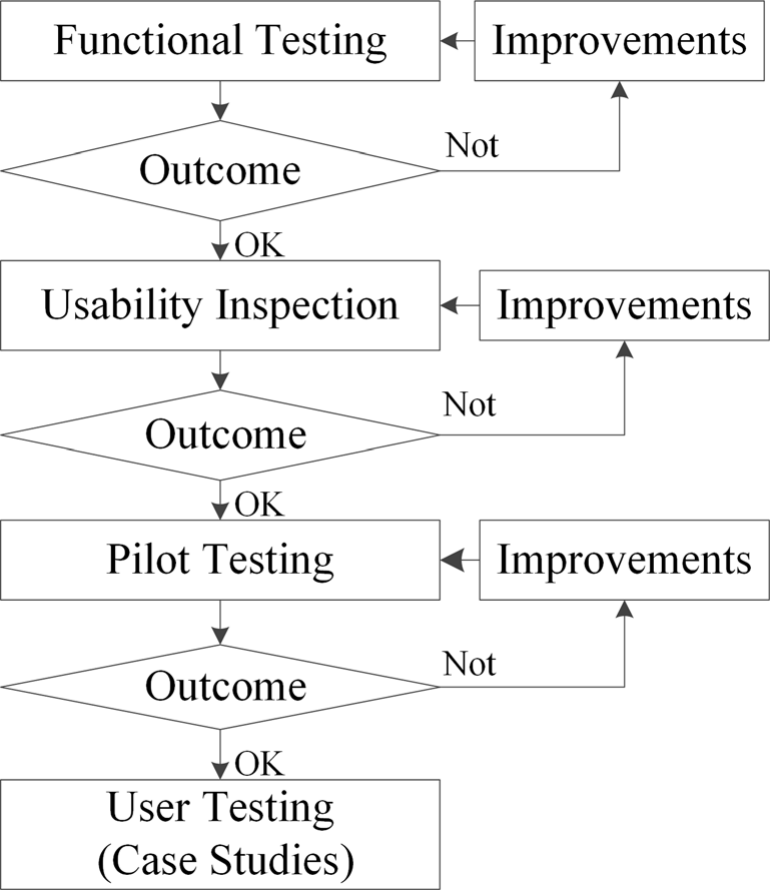
The evaluation methods applied in this research

For a better preparation of user testing, a series of evaluations were performed as shown in Fig. 4. The evaluation started with functional testing. Functional testing refers to the test of computational algorithms to evaluate their stability and accuracy. Only after a satisfactory functional testing, usability inspection was performed. Problems identified in the usability inspection were also reported to the developers. Once the issues were fixed, a pilot study [48] was conducted to: (a) verify the experimental setup and protocols; (b) overcome the learning curve of physicians, especially for using the strokes method and giving input in different orthogonal planes. After testing the protocols, the case studies were performed and measurements regarding the process and result were collected.

### Materials

The pilot study was conducted at the Department of Radiation Oncology, University Medical Center Freiburg, Freiburg, Germany in February 2014. Table 1 presents the materials used for pilot and follow-up case studies. Utilization of the datasets for this study was approved by the Ethics Committee of the University Medical Center, Freiburg. Before the test, a senior physician was asked to manually segment the organs in each dataset and the outcomes were used as the reference standards.

**Table 1:** Materials used in the pilot testing and case studies

	Pilot testing	Case studies	Details
Time	February 2014	May 2014 and August 2014	
Datasets	7 datasets (lung region) who underwent planning CT	5 datasets (lung region) who underwent planning CT	All the five datasets were acquired on a Philips® Gemini TF Big Bore PET/CT. Every scan was taken based on the lung protocol followed in the University Medical Center Freiburg, Germany.
Participants	2 physicians	2 physicians (P1, P2)	Clinicians with 7.5 years and 5 years of experience respectively, both from University Medical Center Freiburg, Germany.
Types of SAS methods	Strokes only	Strokes and contour	
Number of organs to be segmented	Spinal cord, lung, heart, trachea and proximal bronchial tree (5 organs)	Spinal cord, lung, heart, trachea and oesophagus (5 organs)	Each physician contoured 42 (21 + 21) case studies using both methods. Due to time constrains the lung and oesophagus were segmented only in 3 datasets and rest of the organs were segmented in 5 datasets

### Test Setup and Protocol

**Fig. 5:**
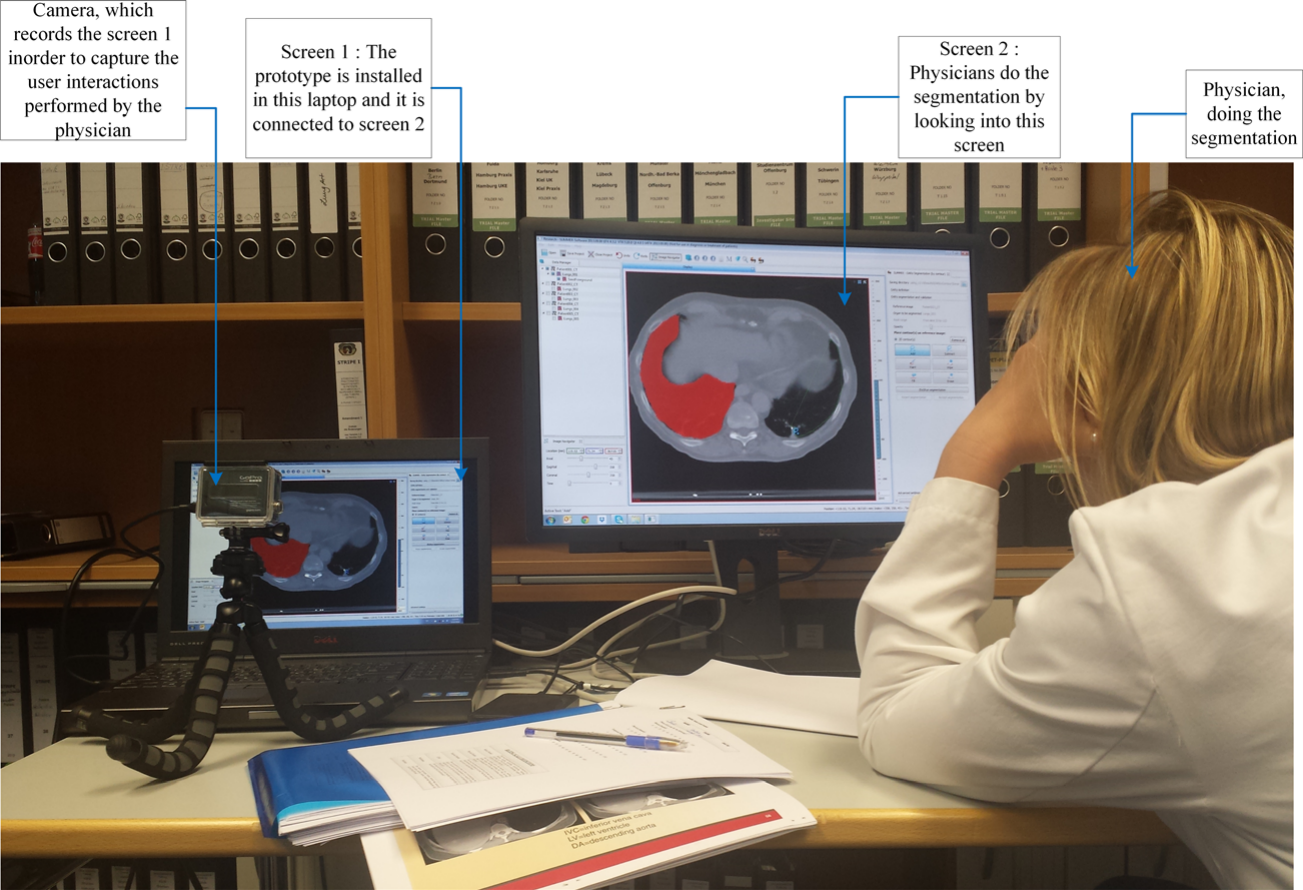
Setup of the user test

The case studies were also conducted at the Department of Radiation Oncology, University Medical Center Freiburg, Freiburg, Germany in May 2014 and August 2014. The same study was conducted twice to assess the reproducibility of the findings. Figure 5 shows the experimental setup. In the experiment, the prototype was installed on a laptop. The laptop display (Screen 1) was mirrored on a 22-inch monitor (Screen 2), which is the screen size that physicians are familiar with. A camera was setup in front of the laptop screen to record the complete interaction process.

Prior to the study, both physicians signed an informed consent form. Subsequently, physicians were informed that this prototype has two SAS methods, and the designed user interactions in the prototype were explained. During the user testing, physicians were given 10 min to get familiarized with the prototype. The sequence of the segmentation task was performed based on the types of organ, i.e., physicians were asked to segment one organ for all the cases using both SAS methods and afterwards, physicians moved to the next type of organ. In case of uncertainty regarding the anatomical extension of the organs, a Radiation Therapy Oncology Group (RTOG) [49] atlas was provided. As the user interface was new for the physicians compared to their daily work, a flow chart of the workflow was provided as well.

### Evaluation Method and Measures

As the main objectives of this study are to identify the relations of the HCI process and the quality of the result, the presented evaluation of the SAS methods focused on two aspects: (1) measurement of process (HCI actions) and (2) measurement of the result. For both of them, subjective and objective measures were deployed.

- Objective measure of the process
  In the evaluation of the HCI process, efficiency was measured from two different aspects: (1) the time taken for performing interaction and (2) the thinking/scrolling time. The time necessary for interaction is related to the physical workload, while thinking or scrolling is related to the cognitive workload of physicians during segmentation. These two measures were identified from the video analysis. From the video analysis, we also acquired data regarding other objective measures, such as interaction patterns. The interaction patterns in this experiment refer to the order of selection of the slice for segmenting, tools selection, and selection of different orthogonal planes. This provided insights whether there were any variations in the interaction patterns and if that variation was associated with the segmentation result.
- Subjective measure of the process
  In the experiment, the NASA-TLX questionnaire was used in each case study to determine the physical demand, mental demand, temporal demand, performance, effort, and frustration of the physician from a subjective point of view. The NASA-TLX [50] is a self-reported subjective technique for assessing mental workload and was developed by NASA.
- Objective measure of the result
  For each type of user interaction, the Dice similarity coefficient (DSC) [51] between the outcome and the reference standard was computed to measure the accuracy of the segmentation result. Dice similarity coefficient is denoted as *S* = 2*c*/(*a* + *b*), where *a* is the volume of segmentation result, *b* is the volume of the reference standard and $c$ is the intersection of *a* and *b*. Besides, the Wilcoxon-signed rank test was also used to find out if there are any statistically significant differences in the result.
- Subjective measure of the result
  A semi-structured interview was conducted at the end of the testing to find out if physicians were satisfied with the result and also to find out about the preference of the two SAS methods.
- Correlations of subjective and objective measures regarding the process and the result
  Table 2 lists the subjective and objective measures that were used in the presented research. To gain additional insights, correlations between the process and the result measures were computed using the Pearson product–moment correlation coefficient. These correlations could be (1) correlated; (2) inversely correlated; or (3) not correlated. This study considered 0.7–0.99 as strongly correlated, 0.4–0.69 as moderately correlated, and 0.1–0.39 as weakly correlated [52].

**Table 2:** Subjective and objective measures of the process and the result

	Objective	Subjective
Process	Drawing time Scrolling/thinking time Use of tools Drawing	NASA-TLX questionnaire (mental demand, physical demand, temporal demand, performance, effort, frustration)
Results	Dice coefficient	Subjective preference

## Results

In this section, the subjective and objective measures of both the process and results are presented. In total, 42 segmentation results from physicians were compared. Out of 42 cases, 18 segmentations were rejected by physicians because of unsatisfactory outcomes. In the rejected segmentations, 14 were using the contour interaction method, while the rest, four were using the strokes interaction method.

### Drawing and Scrolling time of the Strokes and the Contour Methods

The drawing time of both physicians is shown in Table 3. When the two methods were compared against each other for both physicians, lung segmentation showed significant difference in drawing time (*p* = 0.0007, *p* = 0.0001) using the Wilcoxon two sampled test, where the strokes method was much faster than the contour method. Even though there was difference in the mean oesophagus segmentation time for physician 2, there was no statistical difference (*p* = 0.7, *p* = 0.5). It was found that the mean drawing time for physician 2 was always higher than for physician 1 in all the cases with both methods. In addition the contour method took longer time than strokes method in almost all cases.

**Table 3:** The drawing and scrolling time (in seconds) of physicians’ using the strokes and the contour methods

		Physician 1		Physician 2	
Organs		Strokes (s)	Contour (s)	Strokes (s)	Contour (s)
SC	Drawing time	71 ± 10	135 ± 20	135 ± 15	157 ± 40
	Scrolling time	91 ± 30	342 ± 21	151 ± 26	191 ± 51
Lungs	Drawing time	91 ± 8	554 ± 98	95 ± 12	1256 ± 176
	Scrolling time	106 ± 14	116 ± 13	143 ± 10	790 ± 241
Heart	Drawing time	136 ± 15	196 ± 32	209 ± 30	216 ± 31
	Scrolling time	155 ± 19	244 ± 32	143 ± 48	222 ± 33
Trachea	Drawing time	127 ± 21	153 ± 7	184 ± 36	192 ± 43
	Scrolling time	72 ± 34	149 ± 15	162 ± 28	149 ± 49
Oesophagus	Drawing time	258 ± 89	225 ± 56	400 ± 127	300 ± 36
	Scrolling time	193 ± 74	473 ± 29	320 ± 183	434 ± 62

Table 3 shows the average scrolling time of strokes and the contour methods for both physicians, respectively. The scrolling time for segmenting the spinal cord with the strokes method was statistically significant different between physician 1 and 2 (*p* = 0.0002). For the rest of the organs, there was no statistically significant difference in using both methods. When the two methods were compared against each other, the time spent in segmenting the trachea has statistically significant difference for physician 1 (*p* = 0.04) and the time spent in segmenting the spinal cord and segmenting the lung showed statistically significant differences for physician 2 (*p* = 0.03, *p* = 0.008). Even though there was difference in the mean segmentation time for other organs, it is not statistically significant.

### Interaction Pattern

**Fig. 6:**
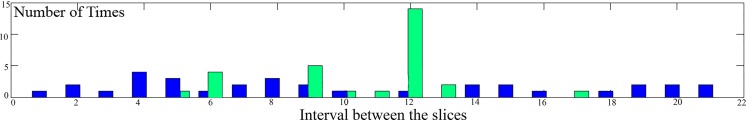
Interaction pattern of the contour method to segment the spinal cord during the initialization

The interaction pattern of contour and the strokes method were analyzed during the first initialization step for both physicians. A consistent drawing pattern was observed for physician 2 in using both methods. For instance, in the use of the strokes method, physician 2 often drew on the first slice, the middle, and the last slices of the dataset. Therefore, the physician only interacted with three or maximum four slices during the first initialization. In using the contour method for heart segmentation, physician 2 contoured in every third or fourth slice, which is similar to the pattern observed in using the strokes method. In the case of segmenting the trachea using the contour method, physician 2 performed interactions in every other fifth slice. Even though physician 1 often started with the center slice, no real pattern could be observed in segmenting the spinal cord, lung, trachea, and oesophagus. In the process of segmenting the heart, physician 1 placed five strokes in almost all the cases. The five strokes were drawn in such a way that the physician started the segmentation on the middle slice in all the cases, moving afterwards to one of the extremes. Figure 6 shows the interaction pattern observed for both physicians of using the contour method in segmenting the spinal cord. In the figure, the horizontal axis represents the intervals between the consecutive slices in which physicians performed interactions, the vertical axis indicates the frequency. The blue bar represents physician 1 while the green represents physician 2. It can be seen in Fig. 6 that almost for every 12 slice intervals, physician 2 performed interactions by drawing the succeeding contours. It was also found that physician 1 did not have a clear drawing pattern and the interactions were performed in random slices.

### Usage of the HCI Tools

The strokes method had only one tool for drawing. Most of the time spent by physicians was related to a consistent usage of this tool, i.e., physicians placed the foreground strokes first and subsequently the background. When advancing to the next slice they started with the background, followed by the foreground to save time. For the contour method, both the “free hand” tool and the “paint” tool are used. The time spent in using the “paint” tool was 3–4 s less than the “free hand” tool in each slice for both physicians. It is also worth mentioning that both physicians needed a certain amount of time to get acquainted with the tools. For instance, physician 1 used the “free hand” tool to correct the segmented boundary where as the “paint” tool was more efficient for this action.

### NASA Task Load Questionnaire

**Fig. 7:**
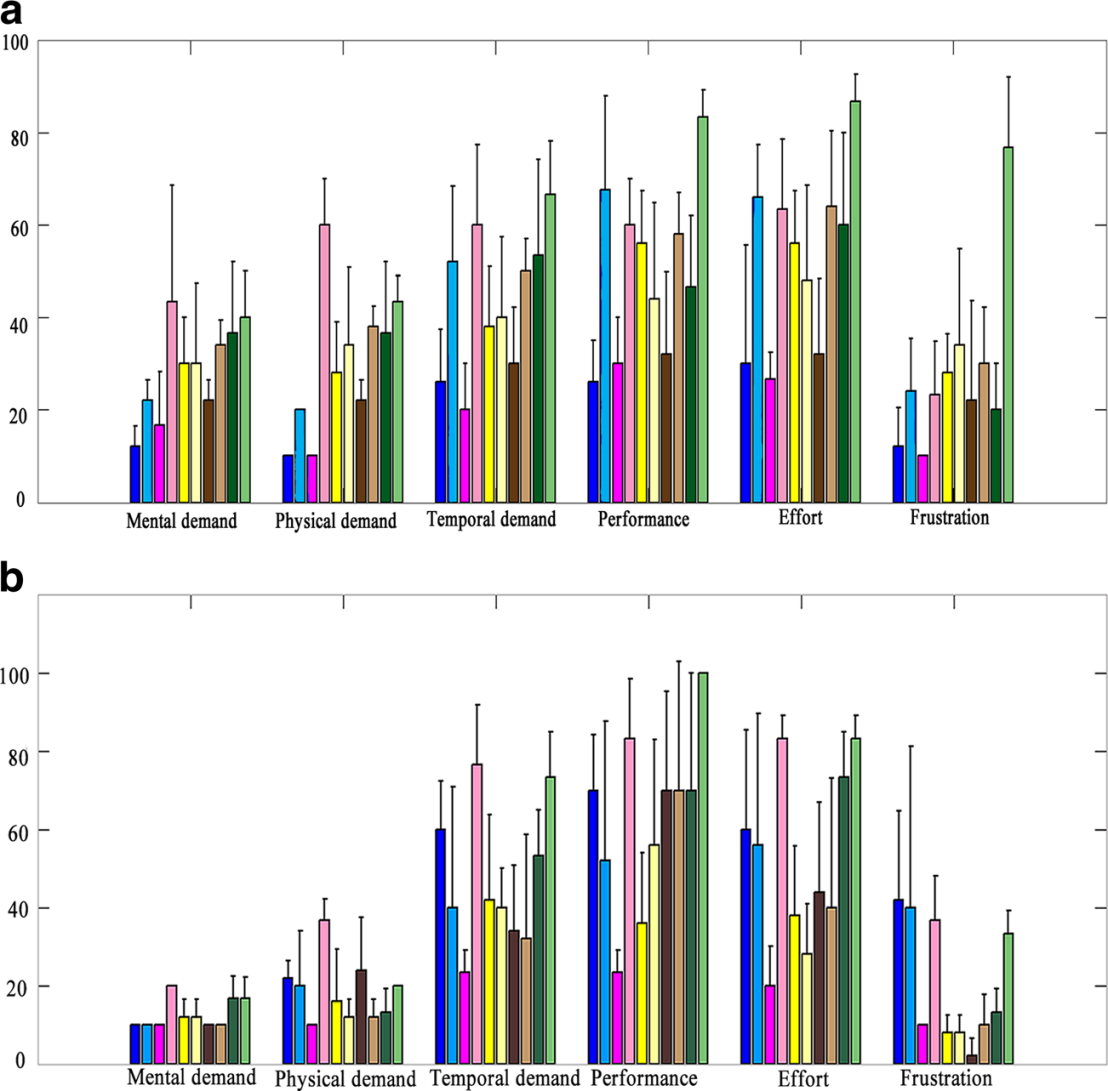
NASA task load of physician 1 (**a**) and physician 2(**b**). Contour method is indicated using *light color* and strokes method using *dark color* for various segmented organs (*blue* spinal cord, *pink* lungs, *yellow* heart, *brown* trachea, and *green* oesophagus)

The NASA task load (NASA-TLX) indices of workload associated with each case study for each physician are shown in Fig. 7. In the figure, the horizontal axis indicates the different types of workload and the vertical axis shows the demand levels ranging from 0–100. For physician 1, the overall workload for oesophagus segmentation was found to be the highest with both methods. For physician 2, the overall workload for segmenting the spinal cord was highest for the strokes method and for the contour method, though the workload for segmenting the lung was highest; there was only a marginal difference over the workload of segmenting the oesophagus.

### Physicians’ Subjective Preference

Table 4 shows the subjective preferences of the physicians for each method in experiment 1. The only difference between experiment 1 and 2 is that physician 2 also preferred the strokes method for the spinal cord in experiment 2.

**Table 4:** Physicians’ subjective preference

Organs	Physician 1	Physician 2
Spinal cord	Strokes	Contour
Lung	Strokes	Strokes
Heart	Strokes or contour	Strokes or contour
Trachea	Contour	Contour
Oesophagus	Contour	Contour

### Dice Similarity Coefficients of the Result

Using the reference standards of each organ, the Dice similarity coefficients of all the organs segmented in experiment 1 are computed as shown in Table 5. P1S indicates physician 1 using the strokes method and P1C refers to physician 1 using the contour method. The Dice similarity coefficients of the spinal cord, the lung, and the heart are higher than 0.8 in almost all cases.

**Table 5:** Dice similarity coefficient of experiment 1

	Spinal cord				Lung				Heart				Trachea				Oesophagus			
Dataset	P1S	P2S	P1C	P2C	P1S	P2S	P1C	P2C	P1S	P2S	P1C	P2C	P1S	P2S	P1C	P2C	P1S	P2S	P1C	P2C
Pt 01	0.89	0.87	0.88	0.87	0.97	0.97	0.72	0.97	0.93	0.93	0.93	0.94	0.61	0.62	0.68	0.62	0.75	0.64	0.44	0.29
Pt 02	0.87	0.86	0.87	0.86	0.95	0.95	0.94	0.94	0.90	0.90	0.90	0.91	0.61	0.63	0.68	0.60	0.66	0.68	0.22	0.47
Pt 03	0.84	0.85	0.84	0.26	0.95	0.96	0.96	0.39	0.93	0.93	0.93	0.94	0.57	0.57	0.69	0.33	0.75	0.69	0.49	0.33
Pt 04	0.88	0.88	0.88	0.88	0.98	0.98			0.93	0.93	0.94	0.90	0.71	0.62	0.48	0.54				
Pt 05	0.90	0.88	0.72	0.89	0.98	0.97			0.95	0.92	0.94	0.58	0.63	0.69	0.73	0.66				

### Correlations

Table 6a–c shows the correlations between measures of the HCI process and performance criteria of the segmentation. For the nine quantitative measures used in the HCI process evaluation, we paired each measure to the others for both types of interaction. A total of 36 pairs were identified for each method. The Pearson correlation coefficient of those pairs is presented in Table 6a, b, regarding the contour method and the strokes methods, respectively. Among those pairs of measures, 20 of contour method and 22 of strokes methods were strongly or moderately correlated, either directly or inversely.

**Table 6:**
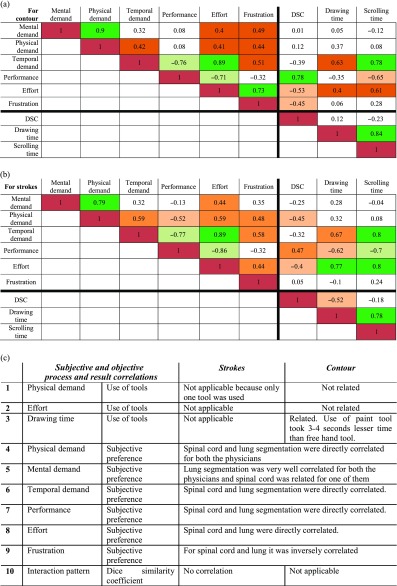
Correlations among different measures in using the contour and the strokes methods

(a) The correlations of using the contour method. Green: strongly correlated, light green: inversely strongly correlated, orange: moderately correlated and light orange: inversely moderately correlated

(b) The correlations of using the strokes method. Green strongly correlated, light green inversely strongly correlated, orange moderately correlated and light orange inversely moderately correlated

(c) List of correlated measures

Table 6c shows the correlations of non-quantifiable pairs. A total of ten non-quantifiable pairs were identified for both interactions. The first three pairs are subjective and objective measures in the process and the remaining seven are paired between measures in the process and the result.

## Discussion

In this study, we identified the impact of user interactions on the segmentation result using two interactive segmentation methods. The user interactions were evaluated subjectively and objectively.

### The Use of Correlated Measures

Table 2 provides both objective and subjective measures for evaluating the interactive segmentation procedure. We correlated those measures and identified the strong, moderate, and weakly correlated pairs. With the paired combinations, it is possible to identify how much effect the designed user interaction has on the HCI process and result. Also the correlated measures provide insights that can be used in improving user interaction design. For example, based on the correlated measures, it was clear that mental demand, physical demand. and temporal demand are correlated to the efforts in both types of interactions and efforts have a direct correlation with frustration. In the use of the contour method, it was observed that frustration and the Dice similarity coefficient are inversely correlated. Hence, efforts and frustration of the users affect the segmentation outcome, as the Dice coefficients represent the quality of the outcome. Thus in future design, the demands of physicians regarding these two aspects should be as low as possible in order to achieve a satisfactory segmentation procedure.

### The Workflow

In the study design, it was assumed that the mental demand is related to the scrolling or thinking time. However, this cannot be verified in this study using the correlations. The physicians indicated that with this prototype they scrolled more than they did in the clinical practice. This could be due to the workflow design. In each iteration of the proposed methods, physicians need to scroll through the entire dataset to evaluate the result and if the result was not satisfactory, they had to scroll again to give the inputs. This should be taken into consideration in the future design of the workflow of SAS methods, as increased mental demands will lead to increased efforts. One way of avoiding this is by showing the result on the current slice and by predicting the result in the next slice. In this way physicians can correct the outcome while scrolling through slices. Another way could be to use the third type of SAS workflows as mentioned in section 2. Using this workflow, the algorithm will re-compute the result only in a small region, rather than re-computing for the whole dataset. It may reduce the processing time of the algorithm and physicians only need to visualize the result in limited regions. However, it will take physicians extra efforts in specifying those “problem” regions.

### The Tools

In the experiment, it was identified that the choice of the interaction tools has some effects on the drawing time, e.g., using the “paint” tool the segmentation time was less than using the “free hand” tool. However, this study did not identify any correlation between the use of a certain tool and the physical demand or efforts. It was also identified that physicians used combinations of different tools while segmenting, for instance, one tool for drawing and another one to adjust the boundary. However, frequent shifting between tools is considered cognitively demanding. Thus, the usage of combined tools may lead to undesired effects, such as an increase in the drawing time and higher mental demand. Hence in the future design, providing a suitable tool for continuous usage is necessary.

### Drawing Pattern

The drawing pattern was another measure which was evaluated in this study. However, only for one physician we identified some systematic drawing patterns. As there was no statistically significant difference on the Dice similarity coefficient we could not conclude that the drawing pattern affects segmentation result.

### Subjective Preference

The subjective preferences of both physicians were the same for all the cases except in segmenting the spinal cord. In segmenting the spinal cord and the lung, the subjective preferences of the physicians were directly correlated with the NASA-TLX indices. For instance, in segmenting spinal cord, physician 2 mentioned that it was easier to draw only contours rather than drawing both the foreground and background strokes. In the case of segmenting the heart, physicians did not give a concrete preference. They felt that with the strokes method they need to increase the size of their paint brush and should contour the whole region in order to get the perfect shape of the organ. Another finding is that when segmenting organs such as the trachea and the oesophagus, physicians always included the cartilage or the organ wall in their clinical routine. In the developed two SAS methods, this was only possible with the contour method. The strokes method was only able to detect the empty volume insides the trachea and the oesophagus. This made the physicians prefer using the contour method for the trachea and the oesophagus, which is different from other OARs. This finding confirms that in the development of the SAS method, physicians should be engaged in the early stage of the development as indicated by Freudenthal et al. [53].

### Differences in Using the Strokes and the Contour Methods

Most of the correlations were nearly the same for using either the contour or the strokes method. However, there are exceptions. One major difference is that drawing time and the subjective performance measure from the questionnaire are strongly correlated for using the strokes method, but not for contour. Also we noticed that the drawing time and efforts are strongly correlated in the use of the strokes method. From Table 3 it can be seen that the drawing time is less for the strokes interaction in almost all the cases except for segmenting the oesophagus. This concludes that the strokes method was more efficient and effective than contour method. However it was mentioned by the physicians during the experiment that the cognitive demand of drawing background strokes are higher than drawing foreground strokes. In some case, this higher cognitive demand shifted their preference from using the strokes method to the contour method.

Different from the study conducted by Yurko et al. [54], our study did not show a strong correlation between mental demand and performance. From Fig. 7, it is clear that the frustration level of the contour methods is always higher than the strokes method. Also the frustration level and the Dice similarity coefficient were inversely correlated in using the contour method. With the inverse correlation and from Table 5, it can be seen that outcomes from the contour method are not as good as the strokes method for all the cases and the mental demand, performance and effort were low in using the strokes method. Hence, strokes can be considered as a preferred interaction in future prototypes.

### Limitations

First, only two participants were included in the study, which limits the study regarding inter-observer variation. Second, due to the novelty of the strokes method, we only introduced mouse as the HCI device. If new input devices were introduced, it would have been difficult to identify the cause of changes in the process and the result. Coen [39] discussed that HCI input devices may also influence the segmentation. Thus, different types of input devices should be considered after physicians are familiar with this method.

## Conclusion

In the proposed research, we investigated the role of user interaction in SAS methods for segmenting the organs at risk in radiotherapy planning. In total, 42 case studies were conducted on five organs with two different SAS methods. Thirty-six quantifiable and ten non-quantifiable correlations were identified for each interaction. Among those pairs of measures, 20 of the contour method and 22 of the strokes methods were strongly or moderately correlated, either directly or inversely. Those correlated measures helped us to confirm that besides the performance of the algorithm, the quality of the segmentation also depends on the physician and the HCI process. Furthermore, the direct and inverse correlated measures provide useful insights for future user interaction design in interactive segmentation. Among the two developed SAS methods, it is clear that the strokes method is more efficient, less cognitively demanding, and requires less effort than the contour method. However it is hard to replace physicians’ subjective preference since cognitively, drawing a contour at the boundary ensures correct segmentation of organs and drawing background strokes was more cognitively demanding. Besides, it is also identified that random and regular drawing pattern did not influence the quality of the result and the duration of the process. These findings suggest that in the future HCI design of SAS methods, user interactions need to be less cognitively challenging and there is a need for flexibility in the interface design.

Current research is directed towards further development of the HCI designs of SAS methods. More HCI devices, for instance, pen-tablet and touch screen, will be introduced to the study. New measures, such as eye tracking, will be introduced as well. The results from the current study will be used to design novel HCI tools in the future studies to improve the effectiveness and efficiency of user interaction.
